# Arecoline-induced EV-mediated ZNF582 hypermethylation drives IFIT1–PD-L1 immune evasion in oral squamous cell carcinoma

**DOI:** 10.1186/s13148-026-02066-4

**Published:** 2026-05-26

**Authors:** Hui-Hsin Ko, Hsin-Hui Peng, Chun-Pin Chiang, Hsiang-Fong Kao, Hung-Ying Lin, Mark Yen-Ping Kuo, Shih-Jung Cheng

**Affiliations:** 1 Graduate Institute of Clinical Dentistry, School of Dentistry, National Taiwan University, Taipei, Taiwan; 2 School of Dentistry, National Taiwan University, Taipei, Taiwan; 3 Department of Dentistry, College of Medicine, National Taiwan University Hospital, Taipei, Taiwan; 4 Department of Dentistry, National Taiwan University Hospital Hsin-Chu Branch, Hsin-Chu, Taiwan; 5 Graduate Institute of Oral Biology, School of Dentistry, National Taiwan University, No. 1, Chang-Te Street, Taipei, 10048 Taiwan; 6 Department of Dentistry, Hualien Tzu Chi Hospital, Buddhist Tzu Chi Medical Foundation, Hualien, Taiwan; 7 Department of Medical Oncology, National Taiwan University Cancer Center, Taipei, Taiwan; 8 Department of Oncology, National Taiwan University Hospital, Taipei, Taiwan; 9 Institute of Oncology, National Taiwan University, Taipei, Taiwan

**Keywords:** Arecoline, ZNF582, IFIT1, PD-L, Oral squamous cell carcinoma (OSCC), Extracellular vesicles (EVs)

## Abstract

**Background:**

This study examined the role of arecoline-induced zinc finger protein 582 (ZNF582) methylation via extracellular vesicles (EVs) in oral squamous cell carcinoma (OSCC) and its effect on PD-L1 expression through the interferon-induced protein with tetratricopeptide repeats 1 (IFIT1) pathway.

**Materials and methods:**

EVs were isolated from SAS and TW2.6 cancer cell lines using ultracentrifugation and characterized using electron microscopy. ZNF582 methylation and protein expression were assessed, with stemness and epithelial-mesenchymal transition (EMT) markers analyzed via Western blotting. T cell populations were evaluated using flow cytometry.

**Results:**

Arecoline-induced ZNF582 methylation via EVs reduced protein expression. ZNF582 knockdown promoted OSCC proliferation, migration, stemness, and EMT, and increased PD-L1 expression, aiding immune evasion via IFIT1. PD-L1 expression was linked to lower CD4+/CD8 + T cell ratios in OSCC patients.

**Conclusion:**

Arecoline-induced ZNF582 hypermethylation via EVs promotes immune evasion through IFIT1, suggesting a potential therapeutic target for OSCC treatment.

**Clinical relevance:**

Targeting the EV-mediated *ZNF582*-*IFIT1*-*PD-L1* pathway may offer new therapeutic strategies for immune modulation in OSCC patients, especially those with a history of areca nut exposure.

**Supplementary Information:**

The online version contains supplementary material available at 10.1186/s13148-026-02066-4.

## **Introduction**

According to 2020 cancer mortality data from Taiwan’s Ministry of Health and Welfare, oral cancer is the fourth leading cause of cancer-related deaths among men, with over 80% of cases linked to betel quid chewing. Oral cancer patients have the youngest average age of death among the top five cancers, highlighting its importance in public health. Despite surgical resection, advanced oral cancer has a local recurrence rate of 40%, and the 5-year survival rate remains at 45%–50%. Improving outcomes requires the exploration of metastasis, drug resistance, and recurrence mechanisms in betel quid-induced oral cancer, focusing on cancer stem cells and the tumor microenvironment (TME).

DNA methylation, a chemical modification of DNA, plays a crucial role in cancer development. This process primarily occurs in cytosine-guanine dinucleotide sequences (CpG islands), where a methyl group is added to cytosine [1]. DNA methylation regulates tumor suppressor and embryonic genes, often accumulating in cancerous or precancerous cells. Analyzing DNA methylation can help detect or predict cancer progression and guide the development of demethylation therapies. Studies have shown that the methylation of genes such as p53, c-Myc, Ras, EGFR, VEGF, PlGF, and SOX9 may contribute to oral cancer development. Additionally, the methylation of five tumor suppressor genes (SOX1, ZNF582, PAX1, NKX6, and PTPRR) is linked to cancer progression, suggesting similar mechanisms in oral cancer [1, 2].

In 1991, zinc finger (ZNF) proteins with a Krüppel-associated box (KRAB-containing proteins) were identified, which encode the Krüppel-type ZNF582, containing one KRAB-A-B domain and nine ZNF motifs. The KRAB-ZNF family likely comprises various physiological processes associated with DNA damage repair, cell cycle control, and neoplastic transformation [3, 4]. Hypermethylated ZNF582 is also found in cervical neoplasms [5].

Our previous research identified ZNF582 gene methylation as a promising biomarker for detecting oral mucosal diseases and predicting oral cancer progression. We observed that ZNF582 methylation levels correlated with disease severity, ranging from normal oral mucosa (NOM) to dysplasia and oral squamous cell carcinoma (OSCC). Moreover, ZNF582 methylation was strongly associated with various oral mucosal diseases, particularly in patients who chewed betel quid alone or with smoking and/or alcohol consumption [6]. Additionally, results from mouthwash-based sampling of oral mucosal cells mirrored those obtained via scraping [7]. Higher ZNF582 methylation in the normal mucosa adjacent to tumors was linked to faster cancer progression and shorter survival times [8]. This suggests that ZNF582 methylation changes occur earlier than visible mucosal alterations and may be more accurate than clinical visual examinations. However, the mechanism by which ZNF582 hypermethylation promotes disease progression remains unclear.

We found that oxidative stress increases extracellular vesicle (EV) secretion, which contributes to cancer development. EVs, lipid bilayer vesicles discovered in the 1970s, are released through various mechanisms such as shedding or budding [9]. Tumor cells secrete EVs, facilitating paracrine and endocrine signaling within the TME. These EVs transport nucleic acids, proteins, and lipids, promoting epithelial-mesenchymal transition (EMT), creating a pre-metastatic niche, and fostering drug resistance, cancer recurrence, or metastasis.

Research has shown [10] that EVs can encapsulate mitochondrial DNA (mtDNA), a circular chromosome encoding 13 proteins essential for metabolism. Unlike nuclear DNA, mtDNA lacks histone protection, making it vulnerable to oxidative stress, which allows it to move into the cytosol. Tumor cells with damaged mitochondria release mtDNA, particularly mtDNA D-loop, into the cytosol, triggering immune responses, such as the production of interferon-stimulated genes. Recent studies have reported that exosomes carrying mtDNA D-loop are closely linked to cancer progression, invasion [11], metastasis [12], and chemotherapy resistance [13].

Additionally, the immune inhibitory protein programmed cell death ligand 1 (PD-L1) can be packaged within EVs and secreted on tumor cell membranes. Elevated levels of EV PD-L1 correlate with tumor progression [14]. EV PD-L1 suppresses T-cell function via regional lymphatic or systemic immune pathways [15]. High levels of circulating EV PD-L1 have been associated with failures in anti-PD-L1/PD-1 therapies [16]. Our previous research showed that under oxidative stress, the mitochondrial Lon protein promoted the translocation of mtDNA D-loop into the cytosol, activating the cGAS-STING-TBK1 pathway. This triggered interferon signaling and led to PD-L1 production. Excessive EV secretion containing mtDNA D-loop and PD-L1 contributed to immune suppression in the TME [17].

However, research on the effect of arecoline, a betel quid alkaloid, on EV production and its contents in oral cancer cells remains limited. No studies have reported how arecoline affects ZNF582 methylation.

This study aimed to investigate whether arecoline influences ZNF582 methylation in OSCC cells. Furthermore, we sought to understand how these changes affect downstream target genes, promoting cancer stem cell characteristics and contributing to immune suppression within the TME.

## Materials and methods

### Patient data and samples

A total of 58 patients with primary OSCC, diagnosed between January 2018 and December 2020 at National Taiwan University Hospital (NTUH), were included in this study, which received approval from the NTUH Research Ethics Committee (reference number: 201811052RINB). All participants provided written informed consent and underwent surgical excision of their primary lesions at the Department of Oral and Maxillofacial Surgery, NTUH. The mean follow-up period from diagnosis to death or the last follow-up was 15 months, whereas the median follow-up period was 14 months (range: 3–37 months). Surgical specimens and blood samples were collected for subsequent experiments. Tumor staging was determined according to the 8th version of the American Joint Committee on Cancer (AJCC) staging system [18]. Demographic and clinicopathological data as well as oral habits were recorded, with regular alcohol drinkers defined as those consuming alcohol more than once per week for at least a year, regular betel quid chewers as those chewing one or more pieces daily for at least a year, and regular cigarette smokers as those smoking 10 or more cigarettes daily for at least a year. Twenty-five participants without oral mucosal diseases, recruited during odontectomy, served as controls (Table 1).

### Cell culture and arecoline treatment

The conditions for cell culture followed previously described protocols [19]. Two stable human OSCC cell lines, SAS and TW2.6 (from tongue and buccal cancers), were used. The cells were cultured in Dulbecco’s modified Eagle’s medium (DMEM; Invitrogen Corporation, Waltham, MA, USA) supplemented with 10% fetal bovine serum (FBS), 100 units/mL of penicillin, and 100 µg/mL of streptomycin, and maintained in a humidified 5% CO₂ atmosphere at 37 °C. To assess the effects of arecoline (Sigma 31593; Sigma-Aldrich, St. Louis, MO, USA), SAS and TW2.6 cells were starved in serum-free medium overnight, followed by treatment with various arecoline concentrations for 0–4 h.

### Isolation and purification of EVs

EV isolation was performed using differential centrifugation techniques outlined in a previous study [17]. In short, the culture medium underwent centrifugation at 300 × g for 10 min to remove cells, followed by spinning at 3000 × g and 10,000 × g to remove debris. The resulting supernatant was centrifuged at 120,000 × g for 90 min to pellet the EVs, which were then washed with phosphate buffered saline. The method mirrored that used for patient blood samples. EV concentration was expressed in µg/µL.

### Transmission electron microscopy characterization of EVs

The morphology and size of EVs were characterized using transmission electron microscopy (TEM), in accordance with previously described procedures. Isolated EVs were fixed in 2% glutaraldehyde in 0.1 M phosphate buffer and stained with 2% phosphotungstic acid solution (pH 7.0). Negative staining was applied to carbon support films, and TEM procedures were conducted using Bio Materials Analysis Technology (Bio MA-Tek, Hsinchu, Taiwan).

### DNA methylation test and DNA methyltransferase inhibitor

Our analysis focused on the ZNF582 promoter region by ISO17025 certified laboratory, specifically targeting its CpG islands, which are critical for gene regulation (iStat Biomedical Co., Ltd, New Taipei City, Taiwan). To ensure accurate results, we employed bisulfite conversion, which converts unmethylated cytosines to uracil while leaving methylated cytosines unchanged. Controls were included to confirm the efficiency and specificity of the conversion process: a positive control (unmethylated DNA) showed near 100% conversion efficiency, and a negative control (untreated DNA) confirmed no background signals. After bisulfite treatment, we conducted quantitative methylation-specific polymerase chain reaction (q-MSP) amplification and sequencing to validate that the analyzed CpG sites were within the promoter region. The primers were specifically designed to amplify the ZNF582 promoter CpG island, and sequencing confirmed our analysis focused on these regulatory regions. These sites are strongly associated with transcriptional regulation, supporting the relevance of our findings [6]. The Cp is the difference of crossing-point (Cp) values between the target gene and COL2A (Cp = Cp_Genem_–Cp_COL2A_). The methylation levels were expressed as the methylation index (M-Index), which was calculated with the formula (2^− Cp^) × 10,000. The DNA methyltransferase inhibitor 5-aza-2’-deoxycytidine (AZC; Sigma-Aldrich) was used for epigenetic modulation.

### Western blotting

Western blotting was performed following standard protocols [20]. Proteins were separated using 10% sodium dodecyl-sulfate polyacrylamide gel electrophoresis, transferred to nitrocellulose membranes, and incubated with specific primary antibodies, including ZNF582, E-cadherin, N-cadherin, IFIT1, PD-L1, Snail, Vimentin, NANOG, SOX2, OCT4a, and GAPDH. Protein bands were visualized using enhanced chemiluminescence (PerkinElmer, Waltham, MA, USA) and quantified using a Fuji LAS-4000 analyzer.

### Cell viability and proliferation assay

Cell viability, proliferation, and cytotoxicity were evaluated using a 3-(4,5-dimethylthiazol-2-yl)-2,5-diphenyltetrazolium bromide-based assay, as previously described [20].

### In vitro migration and invasion assay

Migration and invasion were assessed using a Boyden chamber with 8.0-µm tissue culture inserts. Cells (2–5 × 10⁴) were seeded in serum-free DMEM in the upper chamber, and 2% FBS was added to the lower chamber as a chemoattractant. For invasion assays, OSCC cells (4–10 × 10⁴) were seeded into Matrigel-coated chambers. After 24–48 h, invaded cells were fixed with methanol, stained with 1% crystal violet, and quantified by absorbance at 570 nm.

### Plasmids, transient transfection, and stable clone selection

Transfections were performed using shRNA plasmids targeting ZNF582 and siRNAs for IFIT1 and PD-L1 [21]. Lipofectamine™ 3000 was used for stable clone selection, whereas RNAiMAX was applied for siRNA transfections. Successfully transfected cells were confirmed through Western blotting and used for migration and invasion assays.

### Sphere culture and sphere-forming assay

SAS and TW2.6 cells (2 × 10⁴) were seeded in agarose-coated dishes and cultured for 1–2 weeks. Spheres were counted after being cultured for an additional 7 days, followed by methanol fixation and crystal violet staining.

### Immunofluorescence staining

Cells were fixed in 1% paraformaldehyde and stained with ZNF528, IFIT1 and PD-L1 antibodies, followed by incubation with secondary antibodies conjugated with Rhodamine RedTM. Mounting was performed using Prolong Diamond.

### Enzyme-linked immunosorbent assay and immunohistochemistry

PD-L1 serum levels were measured using enzyme-linked immunosorbent assay kits, and tissue samples were stained for ZNF582 and IFIT1 using the peroxidase-labeled streptavidin-biotin technique. The labeling index was calculated as the ratio of immunostained cells to total cancer or epithelial cells. The specimens were formalin-fixed, paraffin-embedded, and immunostained as previously described [6]. Briefly, antigens were retrieved at 100 °C for 10 min in a 0.1 M citrate buffer after deparaffinized and rehydrated sections of specimens were obtained. H2O2 (3%) was applied to block endogenous peroxidase activity. Sections were then incubated with rabbit anti-ZNF582 polyclonal antibody (1:1000 dilution, GeneTex, Irvine, CA, USA), mouse anti-IFIT1 monoclonal antibody (1:100 dilution, GeneTex, Hsinchu, Taiwan), and rabbit antibodies to PD-L1 (1:1000 dilution, GeneTex), CD4 and CD8 (1:500 dilution, GeneTex) overnight at 4 °C. Sequential incubations were performed with the aforementioned antibodies, streptavidin-peroxidase conjugate, and diaminobenzidine (BioGenex, San Ramon, CA, USA), followed by counterstaining with hematoxylin and examination via light microscopy.

Regarding the quantification of the labeling indices, all counts for ZNF582, IFIT1, PD-L1, CD4 + and CD8+, were carried out using tissue sections at 100x magnification, specifically in areas containing tumor nests. The tumor nests were identified based on histopathological evaluation, and the number of immunostaining-positive cells as a percentage of the total number of counted cancers within these regions. This method ensured that only tumor-associated markers were measured, excluding areas such as normal muscle or stromal tissue.

### Detection and isolation of CD4+/CD8 + T cells via flow cytometry

T cells from OSCC patient peripheral blood mononuclear cells were stained with carboxyfluorescein succinimidyl ester and isolated using a Pan T Cell Isolation Kit. After stimulation with anti-CD3/CD28 and IL-2, T cell proliferation was analyzed via flow cytometry, and CD4 + and CD8 + ratios were evaluated.

### Statistical analysis

Data were expressed as the mean ± standard error of the mean. Two-group comparisons were conducted using Student’s t-test, whereas multiple group comparisons employed a one-way analysis of variance. Survival analysis was performed using the Kaplan–Meier method, with the log-rank test used for group comparisons. A *p*-value of < 0.05 was considered significant.

## Results

### ZNF582 mRNA was negatively correlated with an advanced tumor-node-metastasis stage and poor survival in OSCC patients

**Fig. 1:**
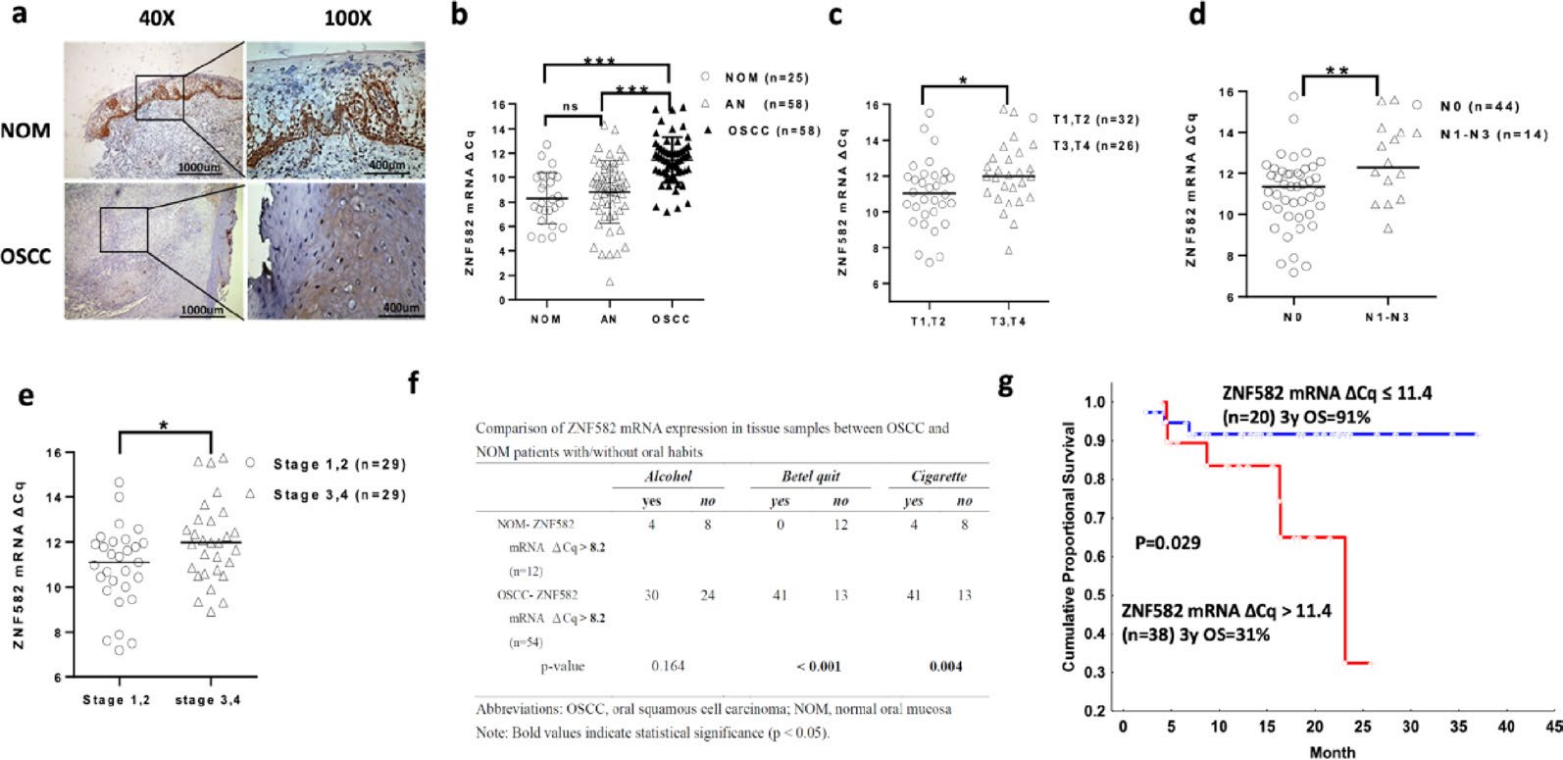
ZNF582 mRNA expression and clinical relevance in OSCC. (**a**) ZNF582 protein staining was strongly positive in the epithelial cells of NOM. By contrast, ZNF582 protein staining was relatively weak in the cytoplasm of the OSCC islands. (**b**) The ZNF582 mRNA △Cq was significantly higher than that in the NOM and AN groups, indicating downregulated ZNF582 mRNA expression in OSCC patients (*p* < 0.001, *p* < 0.001). (**c**) Higher ZNF582 mRNA △Cq was significantly associated with larger tumor size (*p* < 0.01), (**d**) lymph node metastasis (*p* < 0.01), and (**e**) advanced clinical stage (*p* < 0.05). (**f**) ZNF582 mRNA expression was significantly lower in OSCC patients with betel quid chewing or smoking habits than in normal controls (*p* < 0.05). (**g**) The Kaplan–Meier analysis showed that OSCC patients with high ZNF582 mRNA △Cq > 11.4 had significantly shorter overall survival than those with ZNF582 mRNA △Cq ≤ 11.4 (*p* = 0.029, log-rank test)

Fifty-eight OSCC patients (50 men, 8 women, mean age 59 years, range 39–84 years) were analyzed. OSCC cases were localized to the buccal mucosa (33%), tongue (29%), and other oral sites (38%). Tumor-node-metastasis classification revealed 34% stage 1, 16% stage 2, 26% stage 3, and 24% stage 4 (Table 1). ZNF582 protein was strongly positive in NOM but weaker in OSCC (Fig. 1a). ZNF582 mRNA was significantly downregulated in OSCC compared to NOM (*p* < 0.001, Fig. 1b). Higher ZNF582 mRNA △Cq was associated with larger tumor size (*p* < 0.01, Fig. 1c), lymph node metastasis (*p* < 0.01, Fig. 1d), and advanced stage (*p* < 0.05, Fig. 1e). OSCC patients with betel quid chewing or smoking habits had lower ZNF582 mRNA (*p* < 0.05, Fig. 1f). Kaplan–Meier analysis showed shorter survival in patients with ZNF582 mRNA △Cq > 11.4 (*p* = 0.029, Fig. 1g).

**Table 1:** Clinicopathological characteristics of OSCC patients

Patients’ age (years)	Number (*n* = 58)	Number (*n* = 30)
≤ 55	24	12
> 55	34	18
**Patients’ sex**		
Men	50	26
Women	8	4
**Cancer location**		
Buccal mucosa	19	12
Tongue	17	8
Other oral mucosal sites	22	10
**T classification**		
T1 + T2	32	17
T3 + T4	26	13
**N classification**		
N0	44	24
N1 + N2 + N3	14	6
**Clinical staging**		
Stage 1 + 2	29	16
Stage 3 + 4	29	14
**Locoregional recurrence**		
With	11	5
Without	47	25
**Histology of OSCC**		
Well-differentiated	22	11
Moderately and poorly differentiated	36	18
**Risk factors**		
**Alcohol drinking**		
With	32	20
Without	26	10
**Betel quid chewing**		
With	45	22
Without	13	8
**Cigarette smoking**		
With	44	23
Without	14	7

### Arecoline promoted hypermethylation of ZNF582 and inhibited its protein production in SAS and TW2.6 cells

**Fig. 2:**
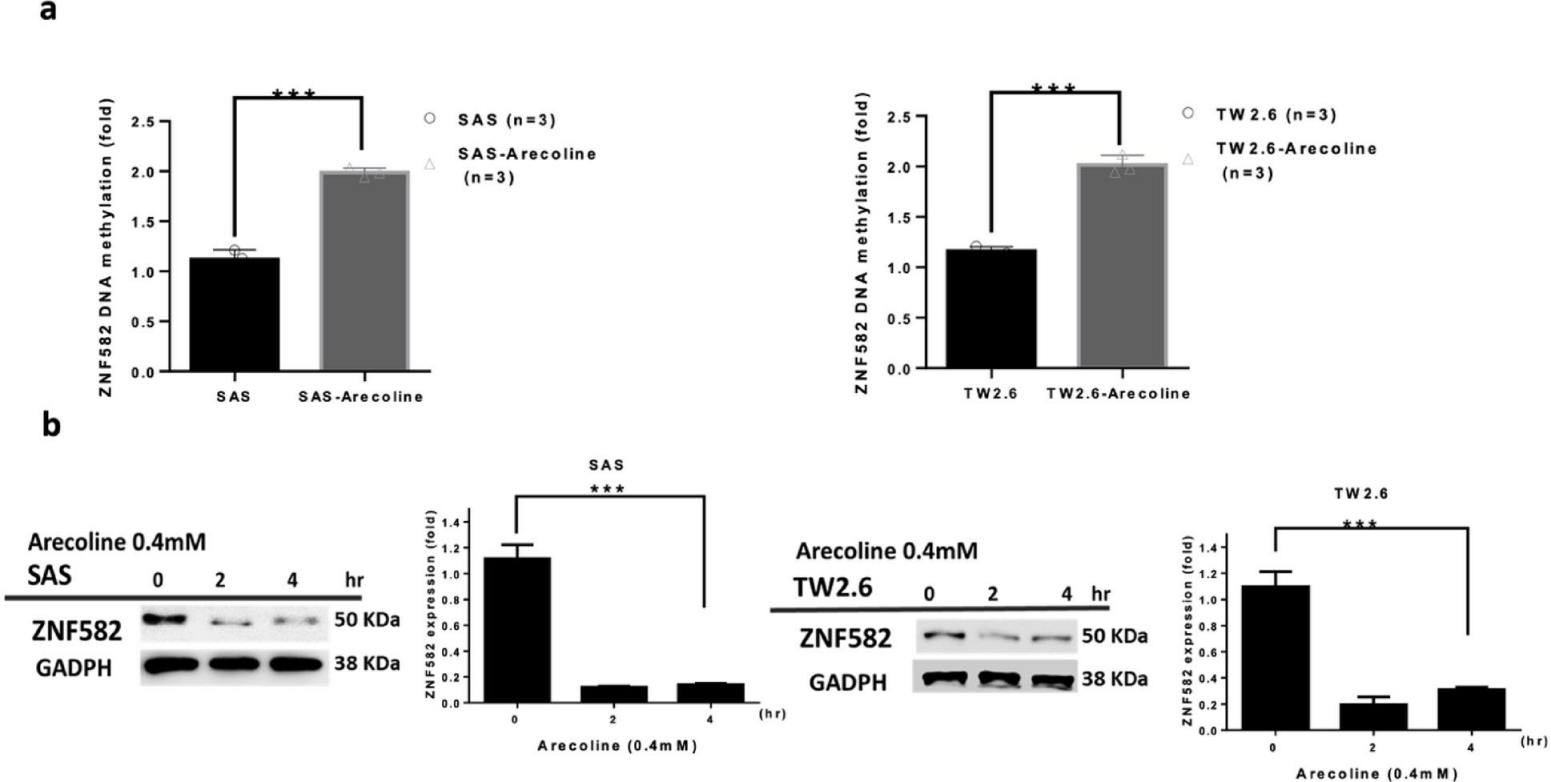
Arecoline-induced ZNF582 gene silencing via hypermethylation. (**a**) Arecoline-induced SAS/TW2.6 cells at 0.1 mM showed higher ZNF582 methylation (*p* < 0.001). (**b**) ZNF582 protein expression decreased significantly with arecoline at 0.4 mM in SAS/TW2.6 cells (*p* < 0.001)

Arecoline treatment at 0.1 mM increased ZNF582 methylation in SAS and TW2.6 cells (*p* < 0.001, Fig. 2a) and significantly reduced ZNF582 protein levels at 0.4 mM (*p* < 0.001, Fig. 2b). Different concentrations of arecoline were applied to separately assess its effects on ZNF582 promoter hypermethylation and protein suppression. A relatively low concentration (0.1 mM) was used in the methylation assay because it represents a physiologically relevant exposure that is sufficient to trigger epigenetic changes without causing significant cytotoxicity, thus allowing us to detect an early effect on DNA methylation. In contrast, a higher concentration (0.4 mM) was necessary to observe a significant reduction in ZNF582 protein levels, as protein suppression typically requires more prolonged or intense exposure. Together, these findings demonstrate a dose-dependent effect: lower concentrations of arecoline induce promoter hypermethylation, while higher concentrations lead to protein suppression. Thus, arecoline promotes hypermethylation of ZNF582, reducing protein production.

### Arecoline increased EV production, promoting ZNF582 hypermethylation in SAS and TW2.6 cells

**Fig. 3:**
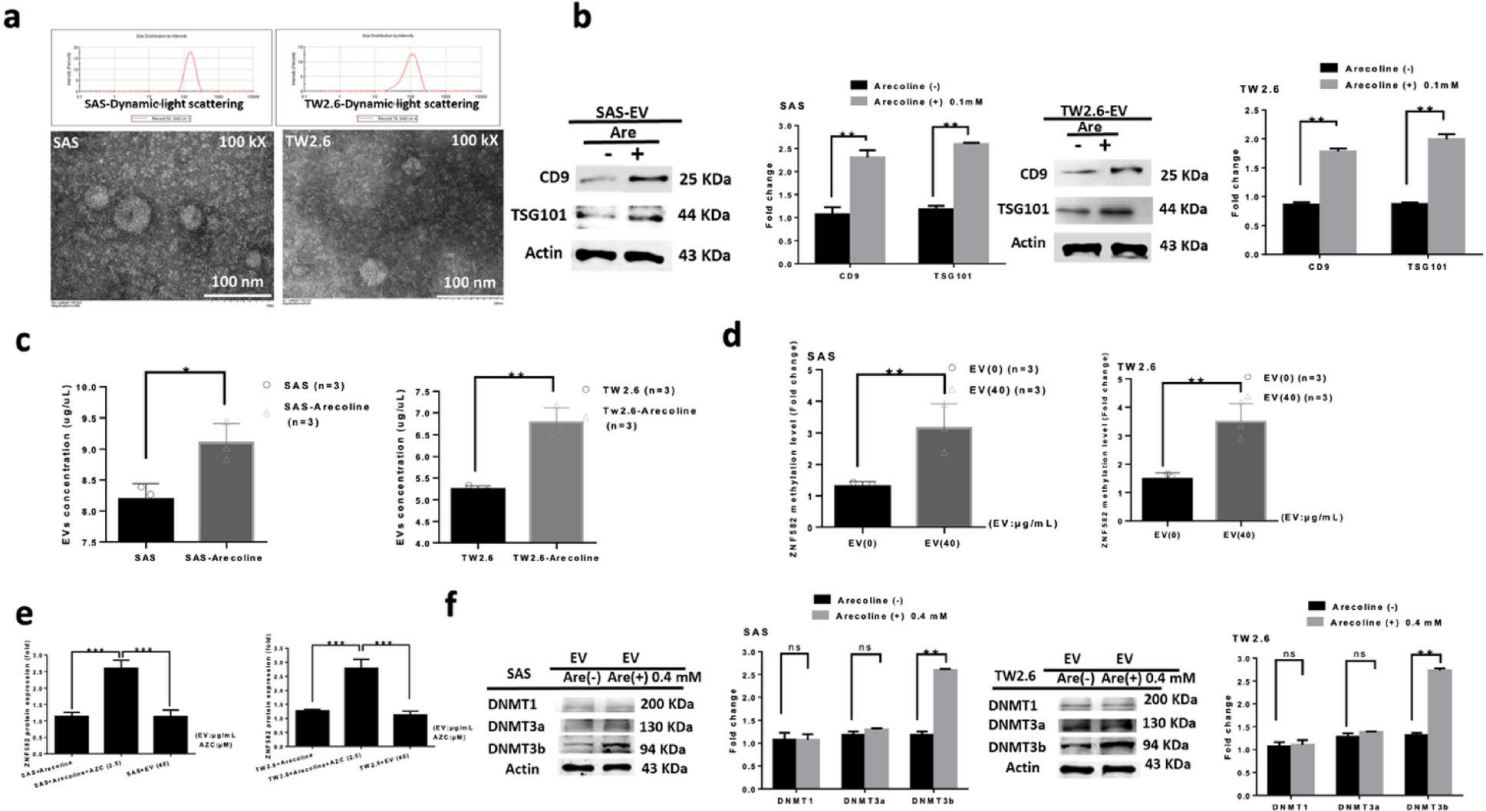
Arecoline-induced EVs promote ZNF582 hypermethylation. (**a**) TEM confirmed the secretion of small EVs (30–100 nm) purified from arecoline-induced cells. (**b**) Western blot analysis identified the EV markers CD9 and TSG101, showing significantly increased secretion induced by arecoline compared to controls (*p* < 0.01). (**c**) Arecoline induction also significantly elevated overall EV secretion compared to controls (*p* < 0.05, *p* < 0.01). (**d**) The methylation level was significantly higher for EV-stimulated SAS/TW2.6 cells than that without EV pretreatment. (**e**) To investigate whether EVs containing DNA methyltransferases (DNMTs) affected ZNF582 methylation, we treated cells with the DNMT inhibitor AZC. We observed that ZNF582 protein expression was significantly reduced with arecoline at 0.4 mM, but this effect was reversed by the DNMT inhibitor AZC. However, EV-stimulated SAS/TW2.6 cells treated with 40 µg/µL of EVs, in the absence of arecoline, exhibited reduced ZNF582 protein expression (*p* < 0.001). These results suggest that arecoline enhances EV production, which in turn promotes hypermethylation of the ZNF582 gene through its effects on SAS and TW2.6 cells. (**f**) Western blot analysis of DNMT1, DNMT3a, and DNMT3b protein levels in SAS and TW2.6 cells were treated with or without arecoline-induced EVs. Arecoline-induced EVs significantly promoted DNMT3b, but not DNMT1 or DNMT3a, protein expression, contributing to ZNF582 hypermethylation (*p* < 0.01; ns = not significant)

Arecoline-induced SAS and TW2.6 cells secreted more EVs, confirmed by TEM and elevated EV markers CD9 and TSG101 (*p* < 0.01, Fig. 3a, b). Arecoline significantly increased EV secretion (*p* < 0.05, Fig. 3c). The methylation level was significantly higher for EV-stimulated SAS/TW2.6 cells than that without EV pretreatment (*p* < 0.01, Fig. 3d). EV-treated SAS/TW2.6 cells showed reduced ZNF582 expression (*p* < 0.01, Fig. 3e), indicating that EVs promote ZNF582 hypermethylation. Furthermore, EVs from arecoline-treated SAS/TW2.6 cells at the concentration of 0.4 mM significantly promoted DNMT3brelative to EVs without arecoline pretreatment, but not DNMT1 or DNMT3a, protein expression, thereby contributing to ZNF582 hypermethylation (*p* < 0.01; ns = not significant, Fig. 3f). These results suggest that arecoline enhances EV production, which in turn promotes hypermethylation of the ZNF582 gene through its effects on SAS and TW2.6 cells.

### ZNF582 regulates EMT and invasion in OSCC cells

**Fig. 4:**
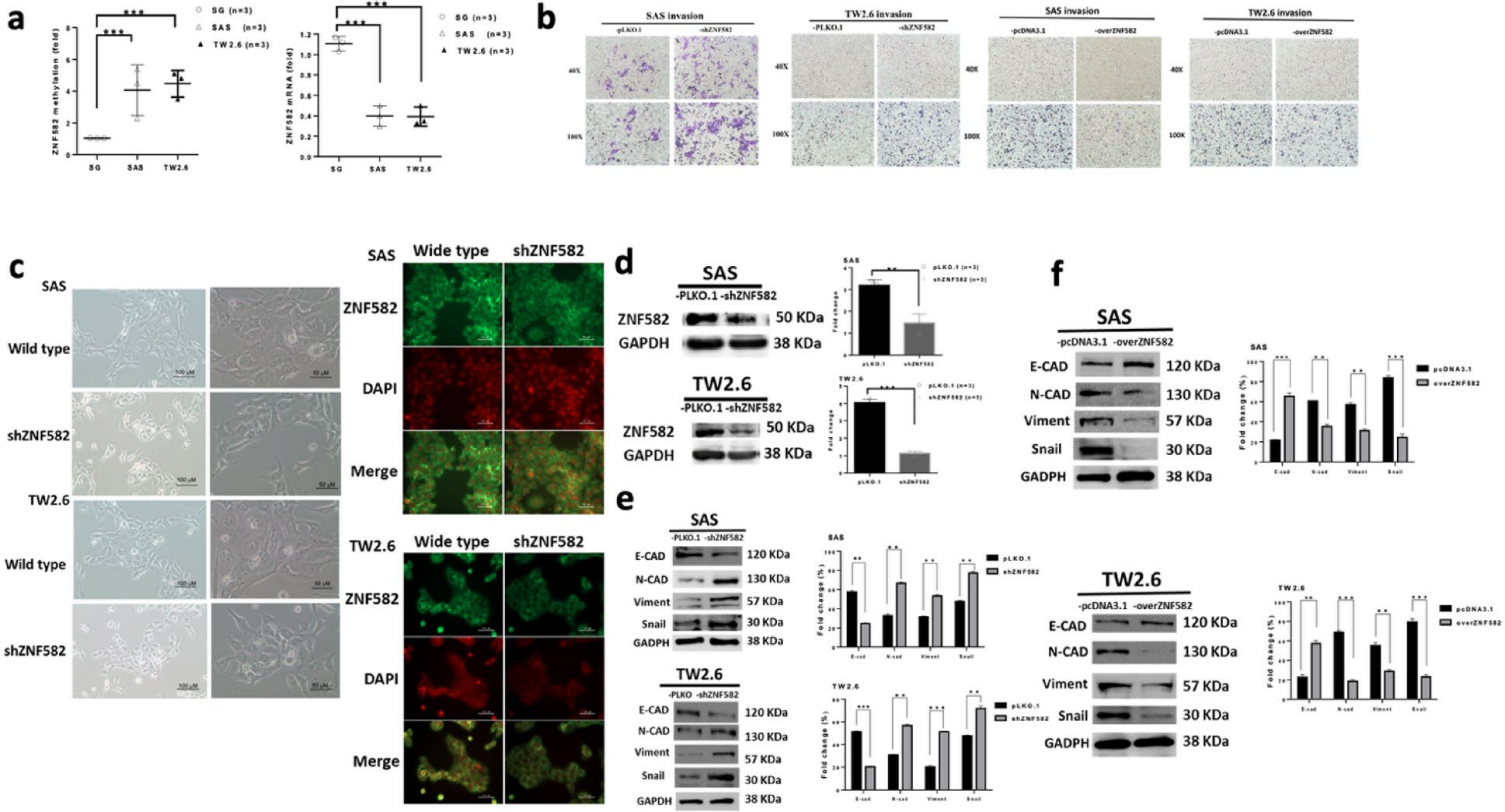
ZNF582 regulates EMT and invasion in OSCC cells. (**a**) In the OSCC cell lines SAS and TW2.6, the methylation level of ZNF582 was significantly higher than that in normal oral cells (SG), (**b**) Functional assays demonstrated that ZNF582 overexpression significantly suppressed cell invasion, whereas ZNF582 knockdown (shZNF582) conversely enhanced the invasion ability. (**c**) In addition, representative phase-contrast unstained and immunofluorescent images showed that there were morphological changes in the shZNF582 cells compared to the control (SAS/TW2.6) cells, including the shZNF582 cells exhibiting more elongated and spindle-shaped morphology, reduced cell-cell adhesion, and a less uniform distribution compared to the control SAS cells. (**d**) Furthermore, shZNF582 SAS/Tw2.6 cells showed significantly reduced protein expression compared with the vector control. (**e**) Knockdown also significantly elevated N-cadherin, Vimentin, and Snail, while reducing E-cadherin in both SAS and TW2.6 cells. (**f**) In contrast, overexpressed ZNF582 significantly reduced N-cadherin, Vimentin, and Snail, while elevating. E-cadherin in both SAS and TW2.6 cells

In SAS and TW2.6 cells, ZNF582 methylation was higher and ZNF582 mRNA expression was lower than in normal cells (Fig. 4a). Functional assays demonstrated that ZNF582 overexpression significantly suppressed cell invasion, whereas ZNF582 knockdown (shZNF582) conversely enhanced the invasion ability (Fig. 4b). In addition, representative phase-contrast unstained and immunofluorescent images showed that there were morphological changes in the shZNF582 cells compared to the control (SAS/TW2.6) cells, including the shZNF582 cells exhibiting more elongated and spindle-shaped morphology, reduced cell-cell adhesion, and a less uniform distribution compared to the control SAS cells (Fig. 4c). Furthermore, shZNF582 SAS/Tw2.6 cells showed significantly reduced protein expression compared with the vector control (Fig. 4d). Knockdown also significantly elevated N-cadherin, Vimentin, and Snail, while reducing E-cadherin in both SAS and TW2.6 cells (Fig. 4e). In contrast, overexpressed ZNF582 significantly reduced N-cadherin, Vimentin, and Snail, while elevating E-cadherin in both SAS and TW2.6 cells (Fig. 4f). These findings indicate that ZNF582 negatively regulates EMT and suppresses the invasive potential of OSCC cells.

### IFIT1 is a downstream target involved in ZNF582-regulated EMT and stemness

**Fig. 5:**
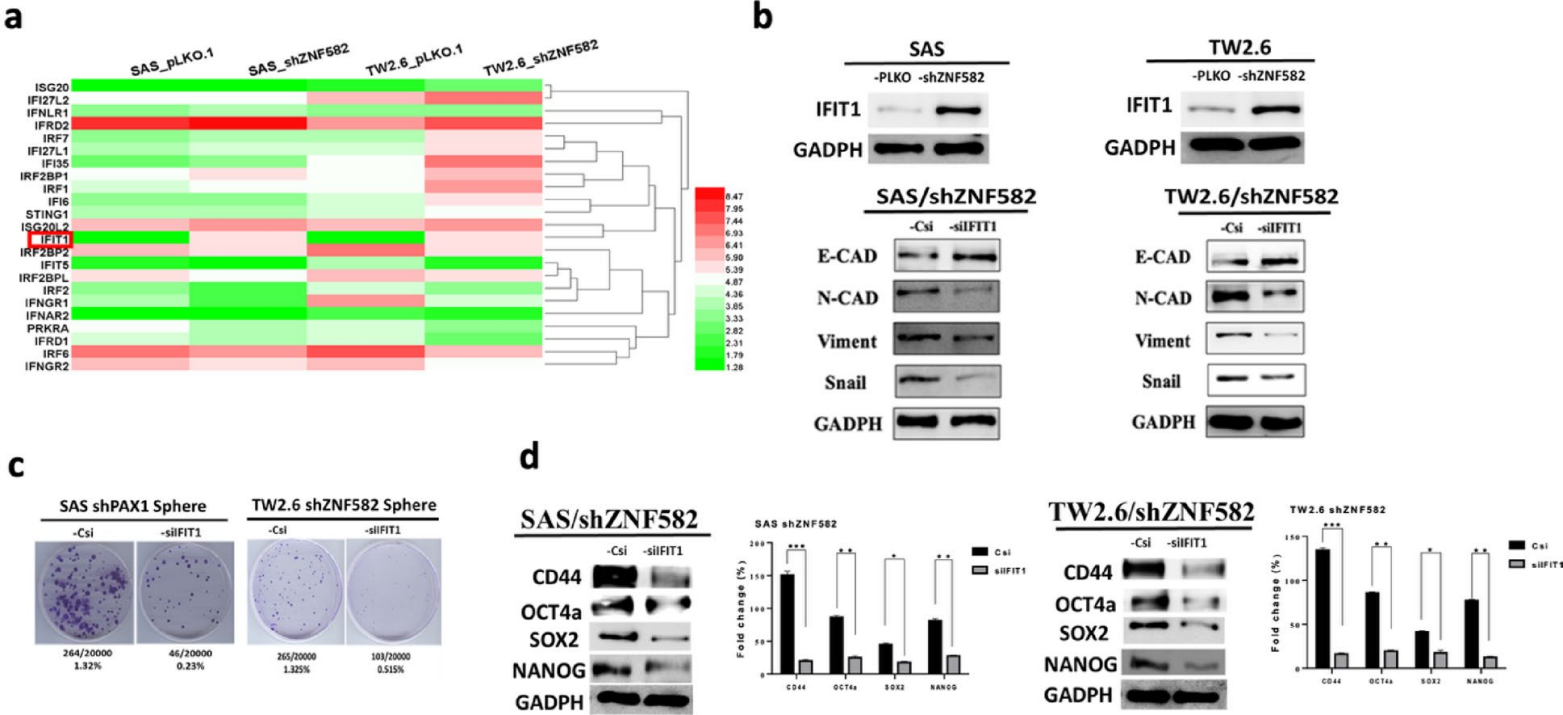
IFIT1 is the downstream target gene involved in ZNF582-regulated EMT and stemness. (**a**) In the OSCC cell lines SAS and TW2.6, analysis following next-generation sequencing (NGS/RNAseq) of wild-type and shZNF582-transfected cells revealed a significant reduction in ZNF582 mRNA expression, with a corresponding increase in the mRNA expression of 92 genes. Further analysis showed that 23 of these genes were IFN-related, with IFIT1 showing the highest upregulation, reaching a five-fold increase, indicating that IFIT1 may be a downstream target of ZNF582. (**b**, upper panel) RNA sequencing results confirmed that IFIT1 expression was significantly elevated in shZNF582-transfected SAS and TW2.6 cells. (**b**, lower panels) Moreover, the upregulation of IFIT1 induced by shZNF582 further promoted EMT and a cancer stem cell-like phenotype. However, when IFIT1 was targeted with siRNA, the expression of Snail1 and N-cadherin decreased, while E-cadherin expression increased (*p* < 0.001). (**c**) siRNA-mediated knockdown of IFIT1 significantly reduced the tumor sphere formation capability induced by shZNF582 (*p* < 0.001). (**d**) Additionally, silencing IFIT1 expression in shZNF582-transfected cancer cells significantly suppressed stemness-related markers (*p* < 0.05, *p* < 0.0001)

Our earlier study [16] unveiled a compelling link between mitochondrial DNA leakage, triggered by mitochondrial Lon protease, and the activation of the STING-IFN axis, culminating in PD-L1 upregulation and immune evasion in OSCC. This revelation spotlighted IFN-related signaling as a critical driver of immunosuppressive mechanisms within the OSCC milieu. RNA sequencing of SAS and TW2.6 cells with ZNF582 knockdown revealed a striking pattern: among the total 15,083 genes, 92 differential expression genes (DEGs) were significantly upregulated > 2 folds in shZNF582 SAS/TW2.6 compared to wild type. However, 23 of which were IFN-related, with IFIT1 showing a five-fold increase (Fig. 5a). IFIT1 expression promoted EMT and stemness, whereas siRNA-mediated IFIT1 knockdown significantly reduced EMT and stemness markers (Fig. 5b, d), confirming its role in ZNF582-regulated pathways. In addition, the results demonstrated a clear reduction in sphere formation upon siIFIT1 treatment, indicating that IFIT1 played a crucial role in maintaining the stemness properties of these cells (Fig. 5c). This supports our hypothesis that IFIT1, which is upregulated following ZNF582 knockdown, contributes to the EMT and stemness pathways in OSCC.

### ZNF582 knockdown promotes PD-L1 expression through the IFIT1 pathway

**Fig. 6:**
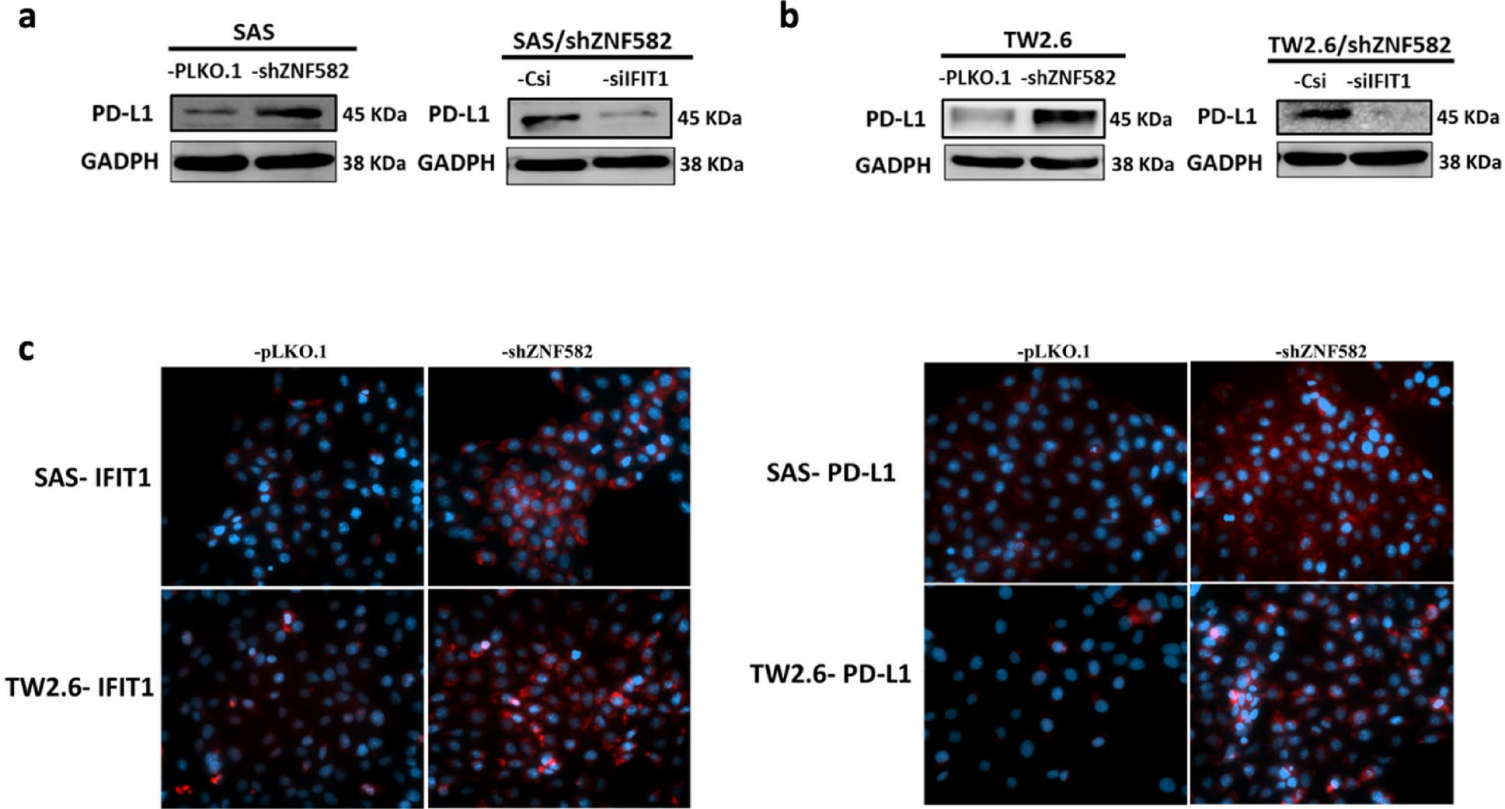
ZNF582 knockdown promotes PD-L1 expression through the IFIT1 pathway. (**a**, **b**, left panels) In OSCC cells, the knockdown of ZNF582 protein promoted the expression of PD-L1 through the IFIT1 signaling pathway. Specifically, in SAS and TW2.6-shZNF582 cells, PD-L1 expression was significantly increased. However, when IFIT1 was targeted and inhibited using siRNA (siIFIT1), the expression of PD-L1 protein was almost completely suppressed. (**c**) Additionally, fluorescence staining results showed that the expression of IFIT1 and PD-L1 proteins (red) significantly increased following the inhibition of ZNF582 protein

ZNF582 knockdown in SAS and TW2.6 cells significantly increased PD-L1 expression (Fig. 6a, b). siRNA-mediated IFIT1 knockdown suppressed PD-L1 expression (Fig. 6a, b). Fluorescence staining showed elevated IFIT1 and PD-L1 proteins after ZNF582 knockdown (Fig. 6c), suggesting that ZNF582 knockdown promotes PD-L1 through the IFIT1 pathway.

### IFIT1 expression and PD-L1 levels correlated with clinicopathological parameters in OSCC patients

Among 58 OSCC patients, we included the clinicopathological characteristics and distribution of the 30 patients analyzed in this subset. As shown in Table 1, the subset of 30 patients is representative of the larger cohort with regard to key characteristics, including age, sex, cancer location, T classification, N classification, clinical staging, histological differentiation, and risk factors (alcohol drinking, betel quid chewing, and cigarette smoking). This ensures that the results derived from the 30 patients are reflective of the broader OSCC population in this study.

**Fig. 7:**
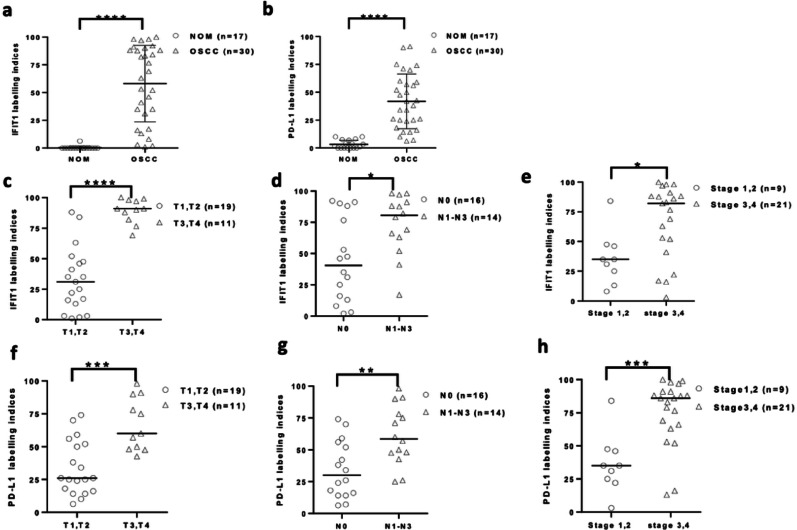
IFIT1 expression and PD-L1 levels were positively correlated with clinicopathological parameters in OSCC patients. (**a**, **b**) IFIT1 and PD-L1 immunostaining PD-L1 were significantly higher in OSCC patients than in NOM (*p* < 0.001). (**c**) IFIT1 expression was significantly positively correlated with large tumor size, lymph node metastasis, and advanced clinical stage (*p* < 0.0001, *p* < 0.05, *p* < 0.05). (**d**) Similarly, PD-L1 expression was significantly correlated with large tumor size, lymph node metastasis, and advanced clinical stage (*p* < 0.001, *p* < 0.01, *p* < 0.001)

In 30 OSCC and 17 NOM samples, IFIT1 and PD-L1 immunostaining were higher in OSCC patients (*p* < 0.001, Fig. 7a, b). IFIT1 and PD-L1 were correlated with larger tumor size, lymph node metastasis, and advanced stage (*p* < 0.05, Fig. 7c, d).

### ZNF582 mRNA expression was negatively correlated with IFIT1 and PD-L1 levels in OSCC

**Fig. 8:**
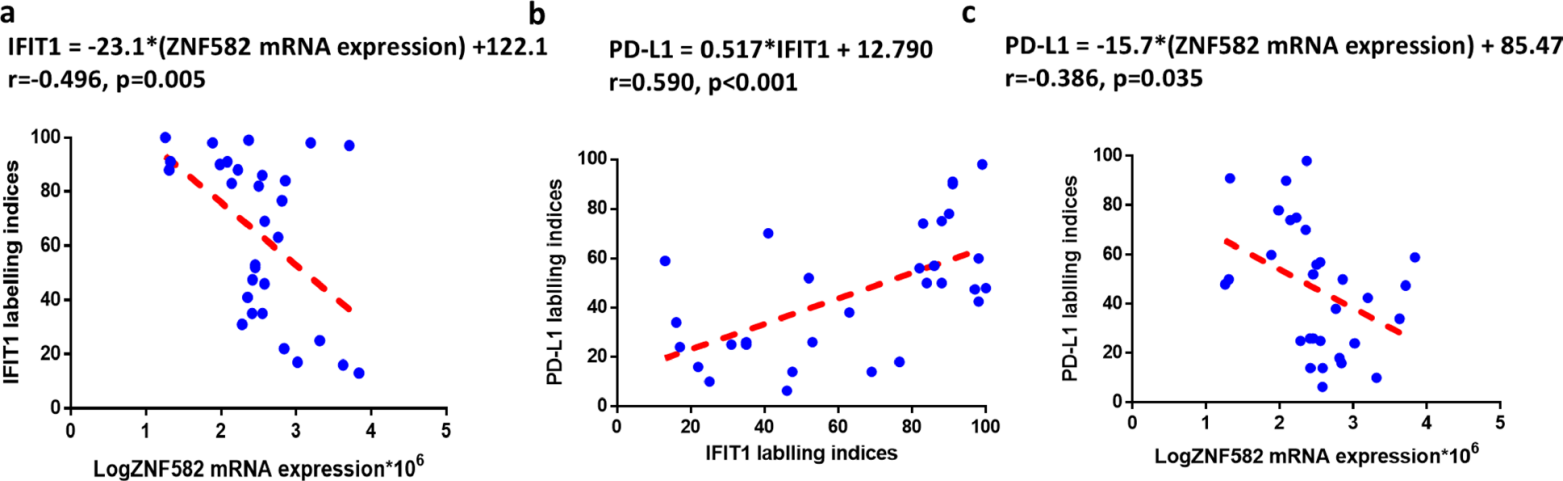
ZNF582 mRNA expression was negatively correlated with IFIT1 expression and PD-L1 levels in OSCC. (**a**, **c**) ZNF582 mRNA expression was negatively correlated with IFIT1 and PD-L1 expression (*r* = -0.496, *p* = 0.005; *r* = -0.386, *p* = 0.035). (**b**) However, IFIT1 expression was positively correlated with the PD-L1 expression (*r* = 0.386, *p* = 0.035)

In 30 OSCC patients, ZNF582 mRNA was negatively correlated with IFIT1 (*r* = -0.496, *p* = 0.005, Fig. 8a) and PD-L1 expression (*r* = -0.386, *p* = 0.035, Fig. 8c). IFIT1 expression was positively correlated with PD-L1 expression (*r* = 0.590, *p* < 0.001, Fig. 8b).

### Impact of CD4 + T cell reduction

**Fig. 9:**
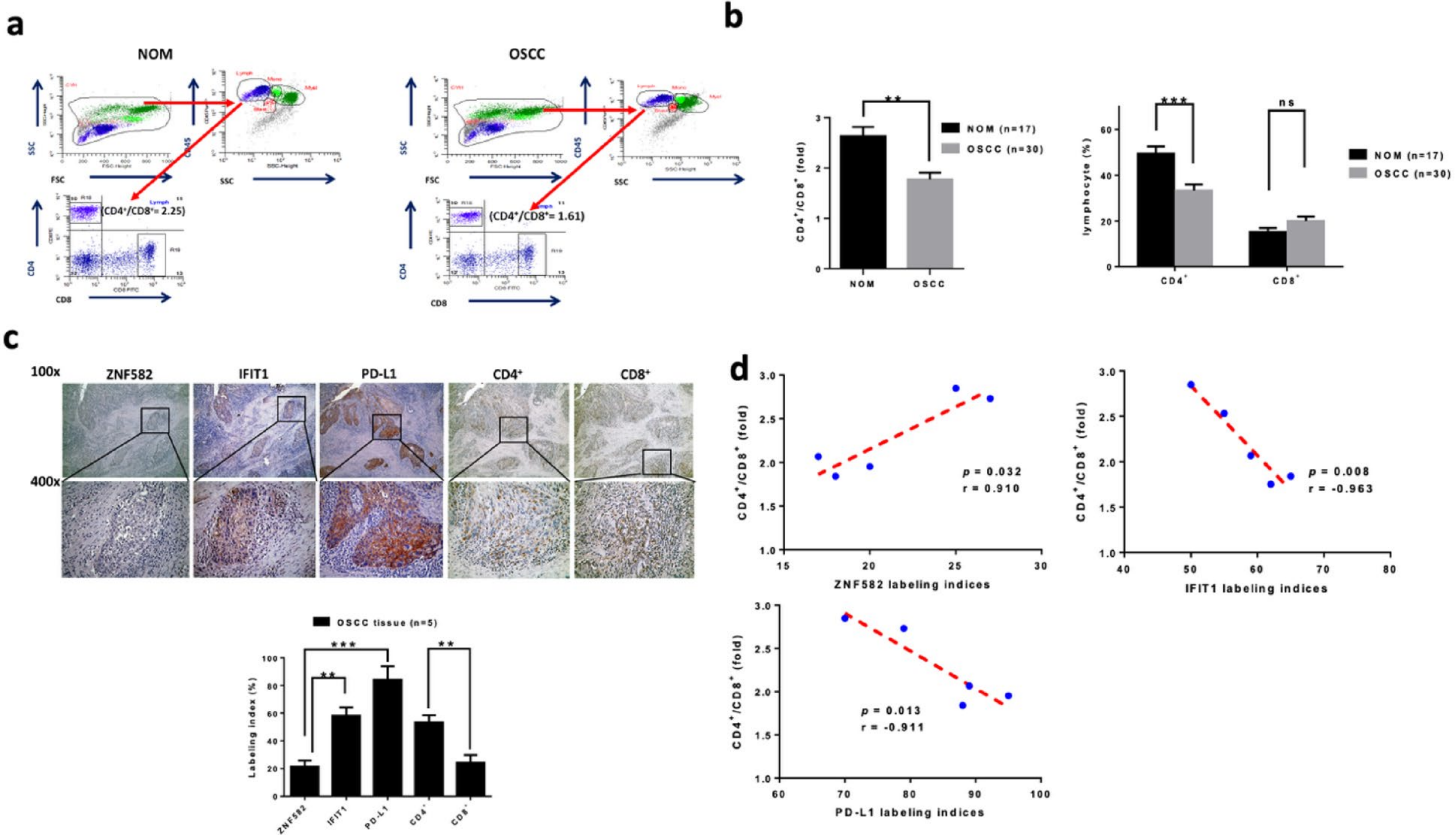
Reduced CD4/CD8 ratio in OSCC linked to ZNF582, IFIT1, PD-L1. (**a**) After whole blood was lysed with the red blood cell lysis buffer, cell pellets were re-suspended and stained in OSCC patients and normal controls and were gated for CD4^+^/CD8^+^ T cell numbers for ratio detection (2.25 vs. 1.61, *n* = 1). (b, left panel) The CD4^+^/CD8^+^ ratio was significantly lower in OSCC patients than in normal controls (*p* < 0.001), (**b**, right panel) with attenuated CD4 + T-cell numbers in OSCC (*p* < 0.0001), whereas CD8 + T-cell numbers did not differ significantly. (**c**, upper panel) Immunohistochemical staining of OSCC tissues for ZNF582, IFIT1, PD-L1, CD4+, and CD8 + markers. Representative images at 100× and 400× magnification show differential expression patterns. (c, lower panel) The bar graph quantifies the labeling indices of these markers in OSCC tissues (*n* = 5) with statistical significance indicated (*p* < 0.01, *p* < 0.001). (**d**) Correlation analysis between the CD4+/CD8 + ratio and ZNF582, IFIT1, and PD-L1 labeling indices in OSCC tissues. A positive correlation was observed between the CD4+/CD8 + ratio and ZNF582 expression (*p* = 0.032, *r* = 0.910), while negative correlations were found with IFIT1 (*p* = 0.008, *r* = -0.963) and PD-L1 (*p* = 0.013, *r* = -0.911) expression, suggesting that higher IFIT1/PD-L1 levels may be associated with a reduced CD4+/CD8 + ratio, potentially reflecting immunosuppressive mechanisms

The CD4+/CD8 + T-cell ratio was significantly lower in OSCC patients than in normal controls (*p* < 0.001, Fig. 9a, b). Reduced CD4 + T-cells in OSCC (*p* < 0.0001) may have attenuated anti-tumor immunity, whereas CD8 + T-cell numbers were unchanged (Fig. 9b). Immunohistochemical staining of OSCC tissues for ZNF582, IFIT1, PD-L1, CD4+, and CD8 + markers. Representative images show differential expression patterns. The bar graph quantifies the labeling indices of these markers in OSCC tissues with statistical significance indicated (Fig. 9c). Correlation analysis between the CD4+/CD8 + ratio and ZNF582, IFIT1, and PD-L1 labeling indices was performed in OSCC tissues. A positive correlation was observed between the CD4+/CD8 + ratio and ZNF582 expression (*p* = 0.032, *r* = 0.910), while negative correlations were found with IFIT1 (*p* = 0.008, *r* = -0.963) and PD-L1 (*p* = 0.013, *r* = -0.911) expression (Fig. 9d), suggesting that higher IFIT1/PD-L1 levels may be associated with a reduced CD4+/CD8 + ratio, potentially reflecting immunosuppressive mechanisms.

## Discussion

Arecoline, a key component of betel quid, promotes oral cancer cell proliferation, progression, and EMT. It induces metastasis by regulating inflammatory cytokines, including serum amyloid A1, IL-6, IL-36G, and chemokine 2. Arecoline also stimulates the production of epidermal growth factor and IL-6, activating the STAT3 and AKT signaling pathways [22].

Recent research, including our own studies, has established that ZNF582 methylation is a reliable biomarker for detecting oral mucosal diseases and predicting disease progression. Specifically, ZNF582 methylation is a critical early screening marker for OSCC [6–8]. However, the mechanisms through which arecoline influences ZNF582 expression remain unclear.

In this study, we observed that arecoline exposure significantly increased ZNF582 methylation in OSCC cells, leading to reduced ZNF582 protein expression. This knockdown of ZNF582 promoted cancer growth and metastasis by increasing PD-L1 expression via the IFIT1 signaling pathway.

EVs, first discovered in the 1970s, are classified into exosomes, microvesicles, and apoptotic bodies, with size ranges of 30–100 nm, 100–1000 nm, and 50–5000 nm, respectively. In our study, we isolated exosomes measuring 30–100 nm from the SAS/TW2.6 OSCC cell lines. These exosomes were characterized by their cellular protein markers, size, and origin [23]. EVs serve as intercellular messengers in the TME and can induce epigenetic changes, including alterations in DNA methylation that lead to carcinogenesis [24]. For instance, microvesicles derived from leukemia cells have been shown to enhance DNA methylation in recipient cells through the activity of DNMT1, DNMT3a, and DNMT3b [25]. Additionally, EVs purified from the gastric fluid of gastric cancer patients have been linked to cancer-related LINE1 and SOX17 DNA methylation [26].

In earlier studies, we demonstrated that oxidative stress in the TME increases EV production via the mitochondrial Lon-ROS pathway. We also found that arecoline induces the production of EVs that encapsulate mtDNA and PD-L1 in OSCC cells, promoting cancer progression [17]. In this study, we confirmed that arecoline-induced EVs further increase ZNF582 methylation, inhibiting the tumor-suppressor role of ZNF582. Prolonged exposure to arecoline-induced oxidative stress disrupts cellular metabolic balance, contributing to oral cancer development.

The three most common epigenetic modifications in cancer are DNA methylation, histone modifications, and non-coding RNA expression [27]. DNA methylation typically occurs in CpG-rich promoter regions, leading to gene silencing without alteration of the DNA sequence [28]. Hypermethylation of tumor suppressor genes can result in carcinogenesis [29]. Histone modifications, such as acetylation and methylation, influence chromatin structure and gene expression in OSCC. For example, histone deacetylase inhibitors have been shown to reactivate silenced tumor suppressor genes and suppress OSCC metastasis [30]. Non-coding RNAs, including microRNAs and long non-coding RNAs (lncRNAs), further regulate gene expression by targeting mRNAs or interacting with chromatin-modifying complexes. MicroRNA dysregulation in OSCC is linked to metastasis and therapy resistance, while lncRNAs such as HOTAIR have been implicated in chromatin remodeling and EMT in OSCC [31]. Therefore, DNA methylation, histone modifications, and non-coding RNAs collectively contribute to OSCC progression. ZNF582 methylation exemplifies how epigenetic alterations can serve as biomarkers and therapeutic targets in OSCC.

Our previous work demonstrated that ZNF582 hypermethylation is linked to the severity of oral leukoplakia and poorer survival rates. We found that ZNF582 mRNA expression was significantly lower in OSCC compared to NOM and abnormal oral mucosa (AN) [32]. ZNF582 mRNA expression was negatively correlated with tumor size, lymph node metastasis, clinical stage, and oral habits, with low expression associated with reduced survival. Data from The Cancer Genome Atlas corroborate these findings, showing high ZNF582 methylation and downregulated mRNA levels in head and neck cancers.

Using two stable cancer cell lines with shRNA knockdown of ZNF582, we assessed their functional capabilities. Compared to the vector control, shZNF582-SAS/TW2.6 cells showed significantly higher proliferation, migration, and invasion. These cells also exhibited increased EMT, characterized by the upregulation of Snail, vimentin, and N-cadherin, along with decreased E-cadherin expression. Additionally, shZNF582 cells had a higher rate of tumor sphere formation and overexpression of stemness markers such as NANOG, SOX2, OCT4a, and CD44. These findings suggest that ZNF582 downregulation enhances cell motility, EMT, and stemness.

To further investigate how ZNF582 regulates EMT and stemness, RNA sequencing was conducted on shZNF582-SAS/TW2.6 and wild-type control cells. The analysis identified 92 differentially expressed interferon-stimulated genes, with IFIT1 showing significant differential expression. Knockdown of ZNF582 significantly increased IFIT1 expression in both cell lines and OSCC tissue samples, indicating that ZNF582 suppression promotes EMT and stemness through the IFIT1 pathway.

The IFIT family, discovered in the 1980s, comprises four members: IFIT1, IFIT2, IFIT3, and IFIT5, all located on chromosome 10q23.31 [33]. IFIT1 and IFIT3 enhance EMT and promote cancer cell migration and invasion, aligning with our findings [34]. Conversely, IFIT2 inhibits EMT, and its higher expression is associated with better prognosis in OSCC patients [35].

Our previous research also showed that oxidative stress activates mitochondrial Lon protein, leading to the release of mtDNA D-loop into the cytosol, which triggers PD-L1 production through the cGAS-STING-TBK1-dependent IFN signaling pathway. This cascade promotes immune evasion by remodeling the TME [16]. In the current study, we found a significant correlation between IFIT1 and PD-L1 expression in OSCC patient tissues, whereas ZNF582 expression was negatively correlated with IFIT1 and PD-L1.

The lower CD4+/CD8 + T-cell ratio observed in OSCC patients highlights an imbalance in the immune system that may weaken anti-tumor immunity. CD4 + T-cells are essential for activating immune responses, and their reduction could impair the body’s ability to control tumor growth. Interestingly, CD8 + T-cell numbers were unaffected, suggesting the imbalance is primarily due to a loss of CD4 + T-cells. This reduction is likely linked to increased PD-L1 expression in OSCC, driven by the ZNF582-IFIT1 pathway. PD-L1 suppresses T-cell activity, particularly CD4 + T-cells, allowing the tumor to evade immune detection and progress. Similar findings in other cancers show that a lower CD4+/CD8 + ratio is associated with advanced stages and worse outcomes in other cancers [36]. In OSCC, this ratio could serve as a useful marker of immune suppression and disease severity. Therapies targeting PD-L1 or restoring CD4 + T-cell function might help improve immune responses and patient outcomes. Future research should explore these approaches further.

In conclusion, this study is the first to demonstrate that ZNF582 methylation suppresses its protein expression, leading to immune evasion through the IFIT1/PD-L1 signaling pathway. However, this study has limitations. The small sample size may have introduced bias, and additional mechanistic studies are needed to clarify whether ZNF582 protein directly interacts with the IFIT1 promoter region. Furthermore, these findings should be validated using in vivo models to confirm the role of the IFIT1/PD-L1 axis in immune evasion.

In summary, arecoline enhances ZNF582 methylation, activating the IFIT1 pathway and promoting PD-L1 overexpression, allowing cancer cells to evade immune surveillance. The ZNF582/IFIT1/PD-L1 axis presents a potential biomarker for molecular targeted therapy in OSCC patients.

## Conclusion

**Fig. 10:**
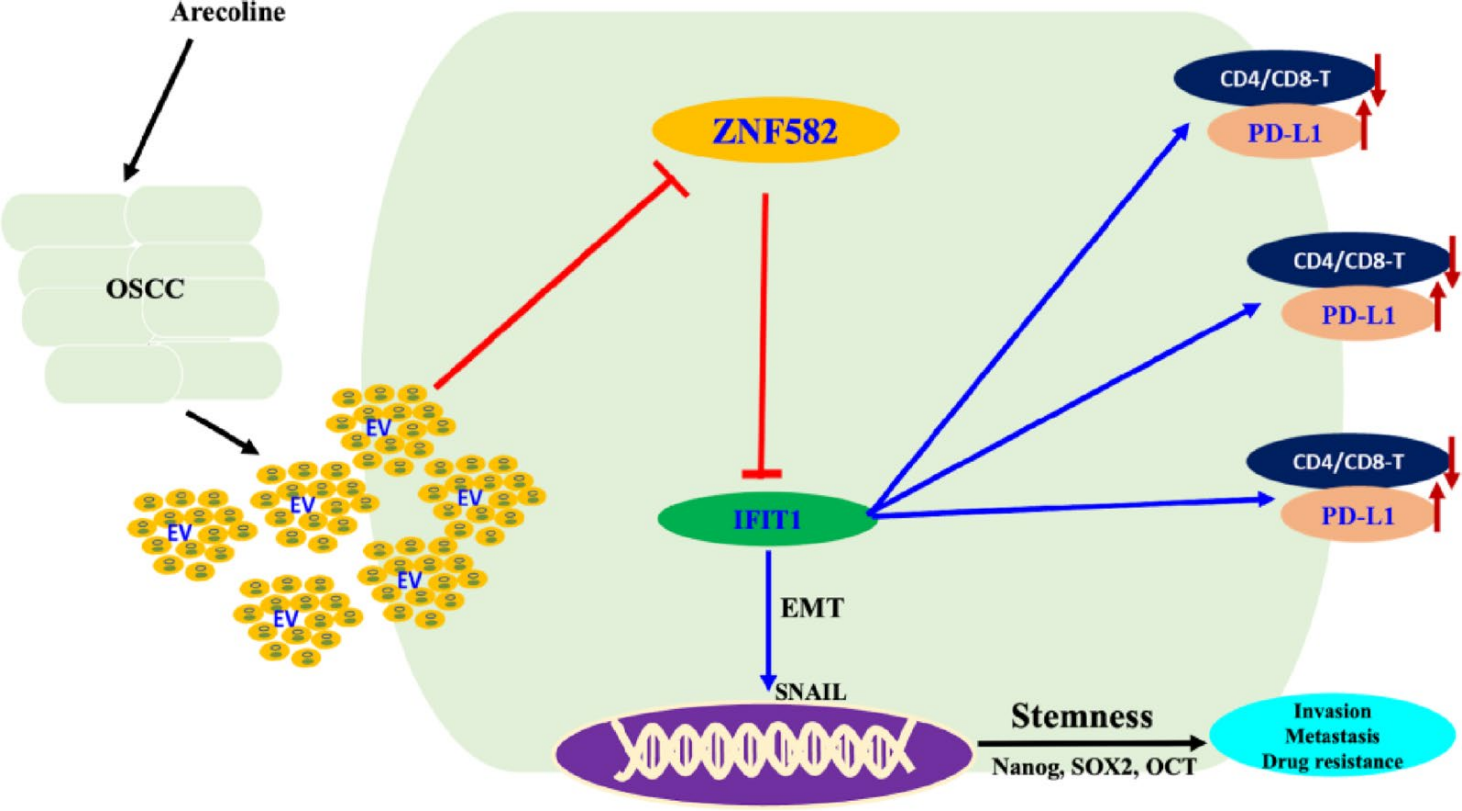
Arecoline-EVs pathway: ZNF582-IFIT1 axis drives OSCC progression

The hypothetical schematic pathway of OSCC cells illustrates that arecoline induces the excessive production of EVs, which results in the suppression of ZNF582 protein synthesis through DNA hypermethylation (Fig. 10). The reduced levels of ZNF582 protein diminish its inhibitory effect on IFIT1, leading to hyperactivation of the downstream IFIT1 pathway. This hyperactivation promotes epithelial-mesenchymal transition (EMT) and enhances stemness. Furthermore, immunosuppression is evident as IFIT1 stimulates the production of the immunosuppressive protein PD-L1 within the TME.

A hypothetical schematic pathway of OSCC cells illustrating that arecoline induces the excessive production of EVs, which results in the suppression of ZNF582 protein synthesis through DNA hypermethylation (Fig. 9). The reduced levels of ZNF582 protein diminish its inhibitory effect on IFIT1, leading to hyperactivation of the downstream IFIT1 pathway. This hyperactivation promotes EMT and enhances stemness. Furthermore, immunosuppression is evident because IFIT1 stimulates the production of the immunosuppressive protein PD-L1 within the TME.

## Supplementary Information

Below is the link to the electronic supplementary material.

Supplementary Material 1

## Data Availability

The datasets supporting the conclusions of this study are publicly available in the Zenodo repository at 10.5281/zenodo.18205108
