# Supplementary figures and images for: Arecoline-induced EV-mediated ZNF582 hypermethylation drives IFIT1–PD-L1 immune evasion in oral squamous cell carcinoma
Source: Clin Epigenetics. 2026 May 26;18:88. doi: 10.1186/s13148-026-02066-4 (PMC13202916; doi:10.1186/s13148-026-02066-4)

## Slide 1
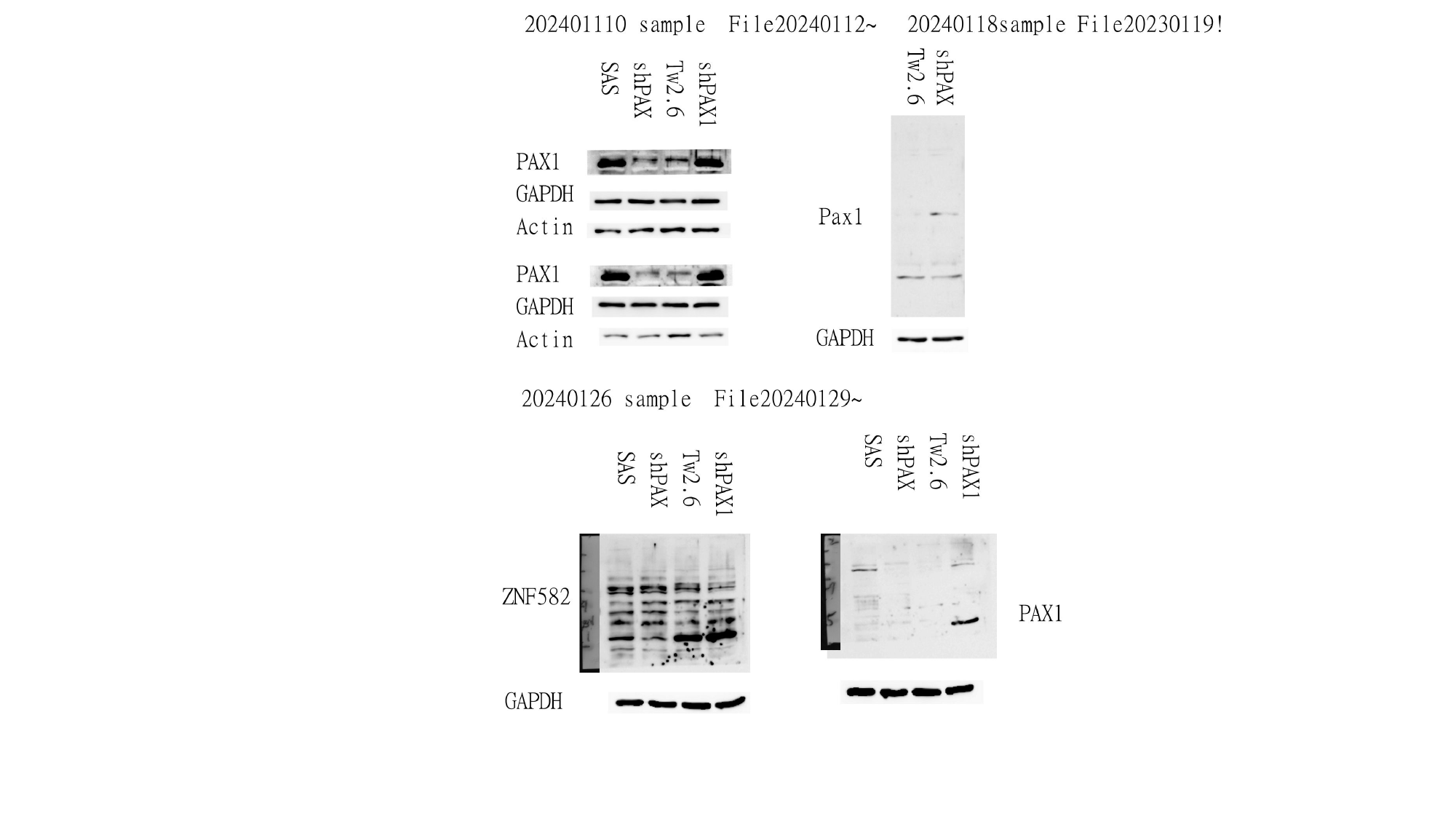

## Slide 2
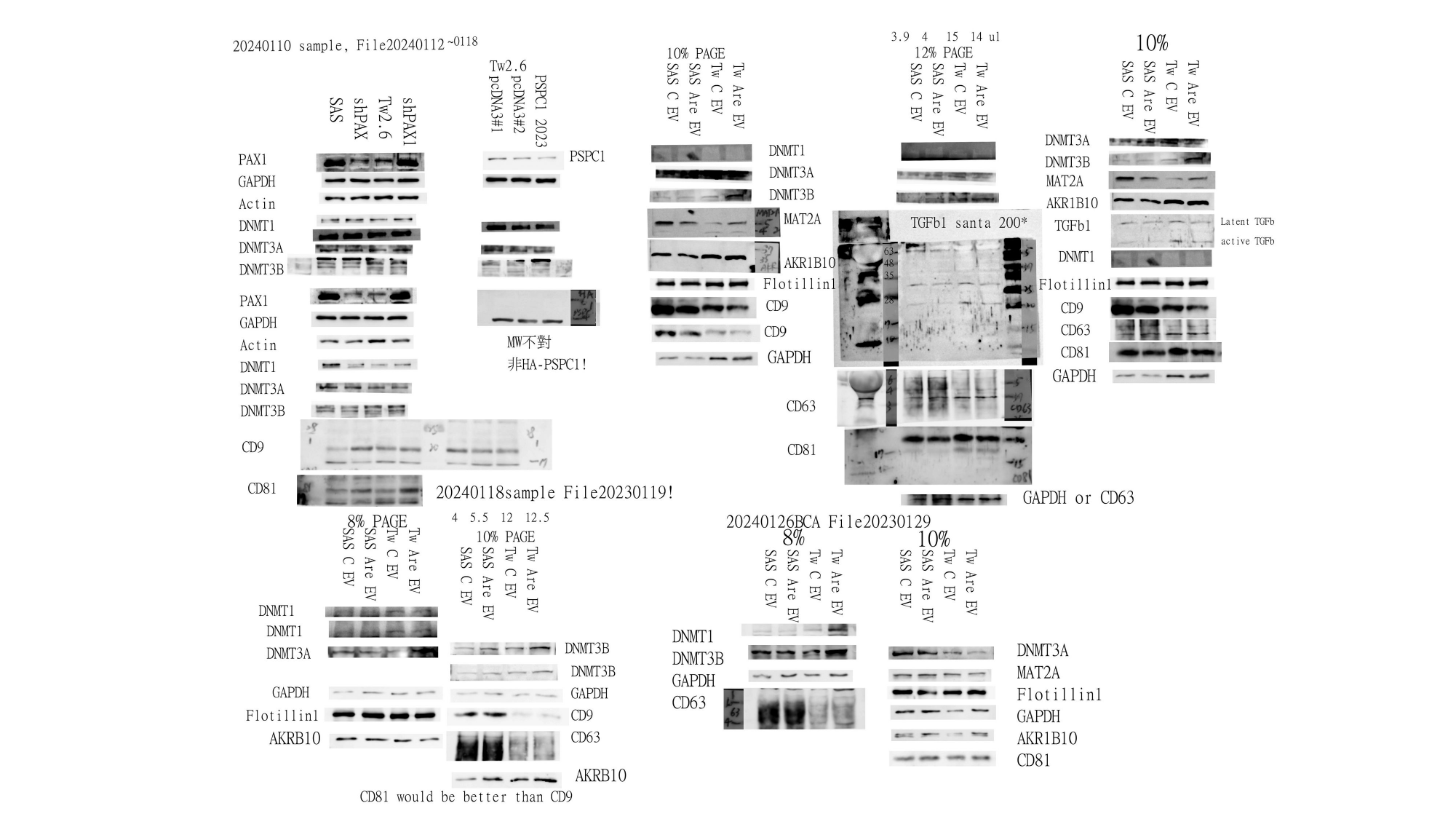

## Slide 3
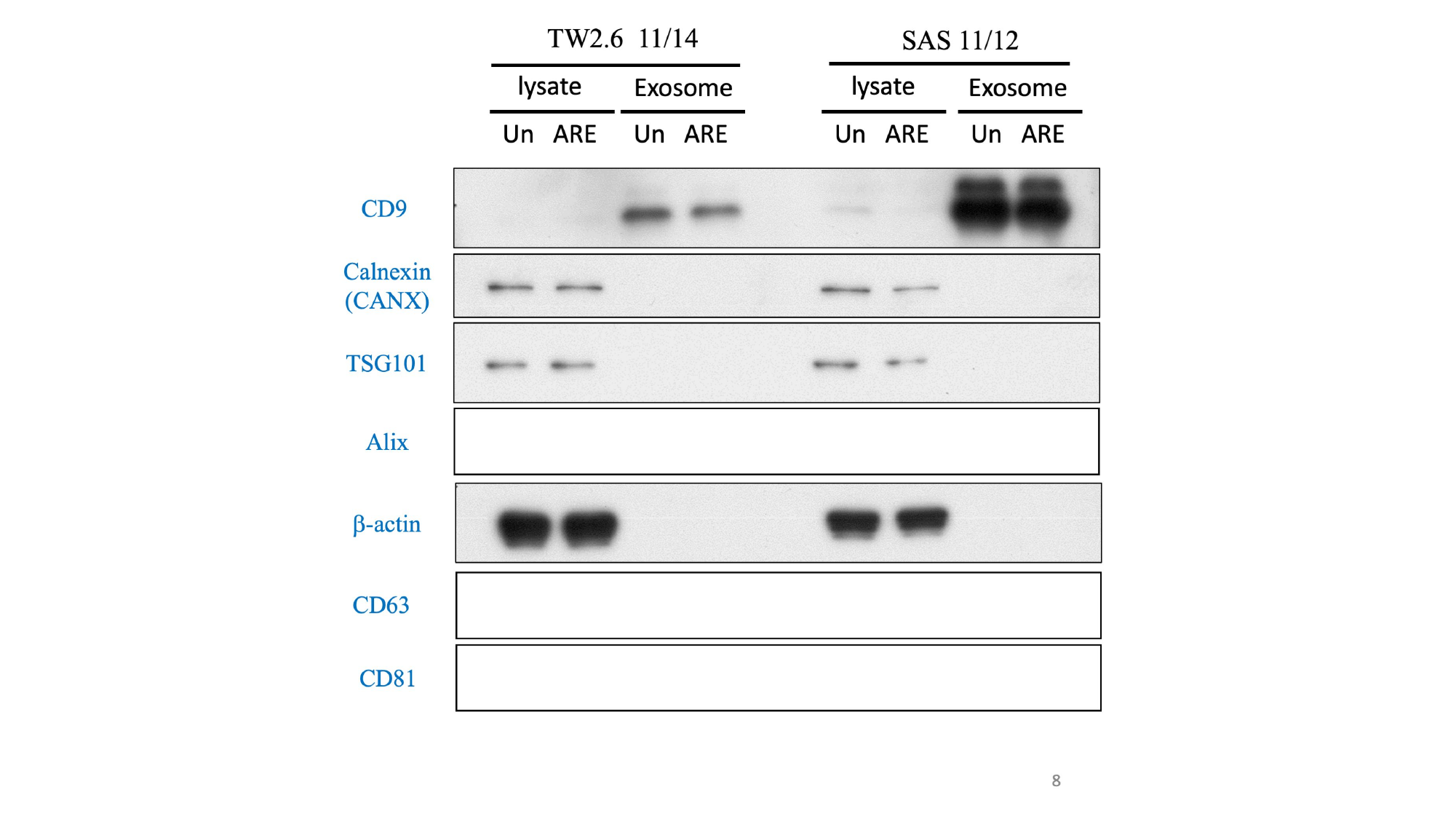

Supplement: Supplementary file 1 — Supplementary Material 1 [file 13148_2026_2066_MOESM1_ESM.pptx]
